# Multiple historical processes obscure phylogenetic relationships in a taxonomically difficult group (Lobariaceae, Ascomycota)

**DOI:** 10.1038/s41598-019-45455-x

**Published:** 2019-06-20

**Authors:** Todd J. Widhelm, Felix Grewe, Jen-Pan Huang, Joel A. Mercado-Díaz, Bernard Goffinet, Robert Lücking, Bibiana Moncada, Roberta Mason-Gamer, H. Thorsten Lumbsch

**Affiliations:** 1 Field Museum, Science and Education, Chicago, 60605 USA; 2 University of Illinois at Chicago, Biological Sciences, Chicago, 60607 USA; 3 Field Museum, Grainger Bioinformatics Center, Chicago, 60605 USA; 4 Biodiversity Research Center, Academia Sinica, Taipei, Taiwan; 5 University of Connecticut, Ecology and Evolutionary Biology, Storrs, 06268 USA; 6 Botanischer Garten und Botanisches Museum, Herbarium, Berlin, 14195 Germany; 7 Universidad Distrital Francisco José de Caldas, Torre de Laboratorios, Herbario, Bogotá, 11021 Colombia

**Keywords:** Phylogenetics, Taxonomy

## Abstract

In the age of next-generation sequencing, the number of loci available for phylogenetic analyses has increased by orders of magnitude. But despite this dramatic increase in the amount of data, some phylogenomic studies have revealed rampant gene-tree discordance that can be caused by many historical processes, such as rapid diversification, gene duplication, or reticulate evolution. We used a target enrichment approach to sample 400 single-copy nuclear genes and estimate the phylogenetic relationships of 13 genera in the lichen-forming family Lobariaceae to address the effect of data type (nucleotides and amino acids) and phylogenetic reconstruction method (concatenation and species tree approaches). Furthermore, we examined datasets for evidence of historical processes, such as rapid diversification and reticulate evolution. We found incongruence associated with sequence data types (nucleotide vs. amino acid sequences) and with different methods of phylogenetic reconstruction (species tree vs. concatenation). The resulting phylogenetic trees provided evidence for rapid and reticulate evolution based on extremely short branches in the backbone of the phylogenies. The observed rapid and reticulate diversifications may explain conflicts among gene trees and the challenges to resolving evolutionary relationships. Based on divergence times, the diversification at the backbone occurred near the Cretaceous-Paleogene (K-Pg) boundary (65 Mya) which is consistent with other rapid diversifications in the tree of life. Although some phylogenetic relationships within the Lobariaceae family remain with low support, even with our powerful phylogenomic dataset of up to 376 genes, our use of target-capturing data allowed for the novel exploration of the mechanisms underlying phylogenetic and systematic incongruence.

## Introduction

With the advent of next-generation sequencing (NGS) technology, the evolutionary relationships of many groups on the tree of life are increasingly resolved and our understanding of the diversification of these groups has been significantly improved^1–3^. However, in many groups, despite the use of NGS data, certain nodes have resisted unambiguous resolution. Conflicting topologies have been inferred from independent NGS data throughout the tree of life. For example, the placement of ctenophores and sponges have proven difficult as some studies place either sponges or ctenophores as sister to all other animals^4,5^. Phylogenomic reconstructions of birds also yielded conflicting relationships for the earliest divergence within Neoaves^6^, perhaps due to inferences from unequal data and taxon sampling: 42 Mbp from 48 bird genomes^7^ versus, 0.4 Mbp from 259 loci sampled from 198 species^8^. In the plant kingdom, inferences from NGS datasets resolve *Amborella* either sister to all other angiosperms^9,10^ or sister to water lilies^11,12^. Similarly, the Gnetales may be sister to pines, all conifers, or all seed plants^13^.

Several reasons have been invoked to explain gene-tree discordance^14^. Gene duplication can cause problems in phylogenetic reconstruction if paralogous loci with different histories are not distinguished within taxa and erroneously analyzed as homologs between taxa^15,16^. Rapid diversifications may lead to the fixation of fewer substitutions and hence to difficulties in resolving phylogenetic relationships. When too many speciation events occur in a relatively short period of time, gene genealogies are not expected to be fully sorted among evolutionary lineages leading to incomplete lineage sorting (ILS). Species tree methods can be used to mitigate the effects of ILS, but computational constraints prohibit fully parameterized methods such as *BEAST^17^ to estimate a species tree directly from hundreds of loci. Instead, species trees for large datasets are estimated from reconstructed gene trees, which can underestimate the support of relationships since these methods use summary statistics or pseudolikelihood^18^. Moreover, reticulate evolution, in the form of hybridization or horizontal gene transfer (HGT), can lead to incongruence among gene trees and obscure phylogenetic relationships^19^. Reticulations are not modeled in the commonly used species tree and concatenation approaches and hence specific, computationally intensive programs are needed to examine whether relationships of organisms are more complex than bifurcation^20^.

Reconstructing phylogenies can be done with nucleotide and amino acid data and these sources of data can yield incongruent phylogenies^21^. Nucleotide data can suffer from substitutional saturation at particular sites in a genome^22^. Such homoplasy is difficult to model in tree reconstruction methods and hence result in phylogenetic signatures being erased^23^. In case of ancient divergences, amino acid sequences may be preferable, since they are less prone to saturation^21^.

Fungi of Lobariaceae (recently also treated as a subfamily within Peltigeraceae^24^) develop conspicuous foliose macrolichens. Nearly 400 species are currently accepted^25^, but the diversity is predicted to reach 800 species^26^. Inferences from variation in three loci failed to resolved either of the three traditional genera (i.e., *Lobaria*, *Pseudocyphellaria* and *Sticta*) as monophyletic, and these were consequently broken up in several genera (e.g. *Crocodia*, *Parmostictina*, *Podostictina* and *Yarrumia*)^27^. Despite the discovery of highly-supported, genus-level clades, the relationships among these new genera remain partially unresolved. None of the phenotypic traits, essential to traditional generic concepts in Lobariaceae, i.e., the presence and type of pores in the lower cortex, defined a clade wherein all descendants exhibit the particular traits: pseudocyphellae and cyphellae no longer define a monophyletic *Pseudocyphellaria* and *Sticta* respectively. These pores have been shown to facilitate gas diffusion into the thallus^28–30^ and may provide an adaptive advantage in temperate environments^31^. Cyphellae likely arose independently in *Dendriscosticta*, which is consistently resolved within the *Lobaria s*.*lat* clade composed of genera lacking pores^27^. Phenotypic characters of the lichen association have been repeatedly shown to be poor phylogenetic predictors for the mycobiont^32–34^, and the newly described genera, *Crocodia*, *Parmostictina*, *Podostictina*, and *Yarrumia*, may represent morphological/ecological chimeric forms between *Pseudocyphellaria* and S*ticta*.

No study has yet critically estimated divergence times in Lobariaceae, but one study^35^ included three specimens from *Sticta*, *Lobaria* and *Pseudocyphellaria* in a fossil calibrated tree of eukaryotes. These samples composed a monophyletic sister group to *Peltigera* diverging roughly 150 million years ago (Mya) and having a stem age of around 70 Mya. However, another study estimated more recent divergences, with the split from *Peltigera* around 90 Mya and a stem age of nearly 50 Mya^36^. Furthermore, two additional studies have estimated divergence times in *Lobaria* and *Sticta*, both reporting a stem age of nearly 30 Mya for each of the genera^37,38^.

We have reassessed phylogenetic relationships in Lobariaceae using 400 target captured nuclear protein coding loci, and a variety of tree inference methods to reconstruct relationships among genera. We sought to assess how historical processes may confound phylogenetic reconstructions and obscure relationships of major lineages within the family. Specifically, we addressed the effect of (1) data type (nucleotides and amino acids), (2) phylogenetic reconstruction method (concatenation and species tree approaches), and (3) missing data. Furthermore, we examined our dataset for evidence of historical processes, such as (1) rapid diversification, and (2) reticulate evolution. Finally, we produced a fossil-calibrated phylogeny to estimate the timing of historical events during the diversification of Lobariaceae.

## Results

### Efficiency of sequencing data recovery and assembly of datasets

Following the read assembly via Hybpiper^39^ we recovered 337.67 of the 400 target genes with 75% coverage. Most coverage (all 400 genes mapped and 398 having 75% coverage) was achieved for *Lobaria pulmonaria* which was used as the reference in HybPiper and for the bait design. The lowest coverage was in *Sticta cinereoglauca* and *Pseudocyphellaria crocata* for which only 386 and 387 targets were recovered and only with partial coverage to the extent that at 75% coverage only 32 and 0 genes were recovered (Supplementary Table S1). This is probably due to the DNA extract quality reducing the hybridization of these sample to the baits. Of the 400 loci sequenced in this study, 138 generated a paralog warnings (an average of 14 paralogs per sample) involving between one and 95 samples (average 11 taxa with multiple sequences at a given locus). The trees produced after running the HybPiper script paralog_retriever.py to obtain all paralogous sequences for all flagged loci exhibited two patterns: (1) the two sequences retrieved would cluster and form a clade or (2) the two sequences would be resolved in two very different clades, separated by a long branch, which is indicative of an ancestral gene duplication event but could also be explained by allopolyploid hybridization (data not shown). In all 138 cases, HybPiper selected only one sequence that had the most sequencing depth and higher percent identity to the target sequence.

### Topological patterns among data types and phylogeny reconstruction methods

**Figure 1:**
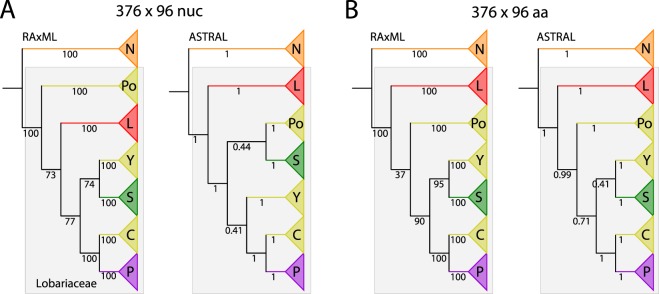
Tree topologies estimated with nucleotide (**A**) and amino acid (**B**) data and different tree reconstruction methods (RAxML concatenated vs. ASTRAL species tree). Nodal support, either as bootstrap (RAxML) or local posterior probability (ASTRAL) is depicted under the branches. Abbreviations are as follows: N = *Nephroma* (outgroup); L = *Lobaria s*.*lat*. clade (also including *Anomolobaria*, *Dendriscosticta*, *Lobariella*, *Lobarina*, *Ricasolia*, and *Yoshimuriella*); Po = *Podostictina*; S = *Sticta*; Y = *Yarrumia*; C = *Crocodia*; P = *Pseudocyphellaria*.

Initially we generated a nucleotide dataset (376 × 96 nuc; Fig. 1A and Table 1) where all included loci had at least 50% specimen representation (at least 48 sequences regardless of recovered length in HybPiper), which was the case for 376 of the 400 loci. In both RAxML and ASTRAL analyses, Lobariaceae formed a well-supported (100% bootstrap support) clade but some of the backbone nodes were poorly supported (Fig. 1A). In the concatenated RAxML analysis, the first split gave rise to samples of *Podostictina*, forming a sister-group relationship to the remaining Lobariaceae. The next split reflects the divergence of *Lobaria* and its closely related genera (*Anomolobaria*, *Dendriscosticta*, *Lobariella*, *Lobarina*, *Ricasolia*, and *Yoshimuriella*). The following split gives rise to the clade composed of two sister genera, *Sticta* and *Yarrumia*. The most derived split among genera yields *Crocodia* sister to *Pseudocyphellaria* (RAxML tree; Fig. 1A).

**Table 1:** Summary of the datasets used in this study.

Dataset	# loci	# tips	Positions	missing data
***nucleotide data***				
397 × 96 nuc	397	96	438036	13.75%
297 × 17 nuc	297	17	338019	0.00%
***amino acid data***				
397 × 96aa	397	96	145467	6.34%
297 × 17 aa	297	17	112265	0.00%

**Figure 2:**
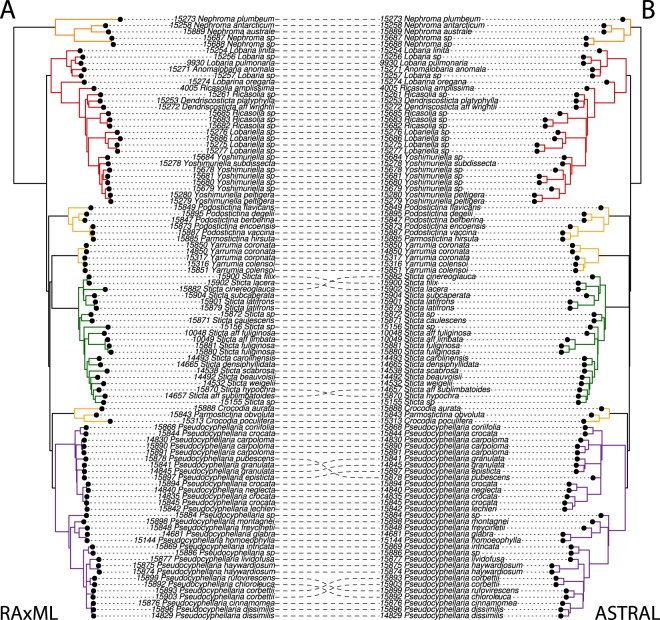
Congruence of topologies inferred with concatenation (**A**: RAxML) and species tree approaches (**B**: ASTRAL) from dataset composed of 376 amino acid sequences. The colors of branches are assigned to clades as follows: Orange: *Nephroma* (outgroup); Red: *Anomolobaria*, *Dendriscosticta*, *Lobaria*, *Lobariella*, *Lobarina*, *Ricasolia*, *Yoshimuriella*; Yellow: *Crocodia*, *Parmostictina*, *Podostictina*, and *Yarrumia*; Green: *Sticta*; Purple: *Pseudocyphellaria*.

Subsequently, we analyzed amino acid sequences from the same dataset (376 × 96 aa; Figs 1B and 2). Again, the calculated tree supported Lobariaceae as a well-supported monophyletic clade, but the RAxML tree had even lower backbone support (37%) for the unique ancestry to the *Podostictina* clade, and the clade including *Sticta*, *Yarrumia*, *Crocodia* and *Pseudocyphellaria*. (Figs 1B and 2).

The species tree inferred in ASTRAL with nucleotide sequences conflicted with the same dataset reconstructed from concatenated data in RAxML (376 × 96 nuc; Fig. 1A), while the amino acid species tree produced in ASTRAL had the same backbone topology as the concatenated amino acid tree (376 × 96 aa; Figs 1B and 2B). However, in the amino acid tree reconstruction from ASTRAL, two nodes were poorly supported; one associated with the splitting of *Podostictina* (0.71 local posterior probability) and another associated with the branching of *Sticta* and *Yarrumia*, both still forming a monophyletic clade (0.41 local posterior probability). The nucleotide species tree differed from the amino acid species tree, with *Yarrumia* being sister to *Crocodia* and *Pseudocyphellaria* with poor support (0.41 local posterior probability) and with *Podostictina* being sister to *Sticta* albeit also with poor support (0.44 local posterior probability; Fig. 1A). *Crocodia* was paraphyletic because *Parmostictina obvoluta* (DNA# 15843) clustered in this clade. *Parmostictina* is polyphyletic, with samples clustering in clades containing samples of *Crocodia* and *Podostictina* (Fig. 2).

### Evidence for rapid diversification and gene-tree discordance

**Figure 3:**
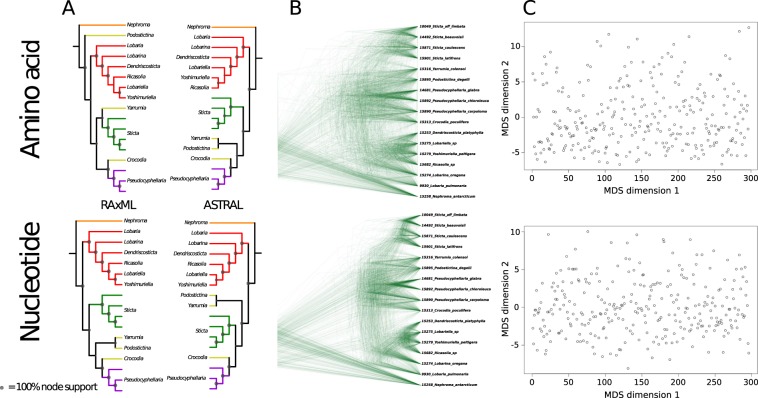
Poor support, incongruence of relationships of genera and gene tree discordance in Lobariaceae with (**A**) different methods (RAxML vs. ASTRAL) and different datatypes (amino acid vs. nucleotide) of the reduced dataset of 297 loci and 17 tips with no missing data. Nodal support is depicted by the size of the gray circles in the nodes, with larger circles being higher supported. (**B**) DensiTree plots of the gene trees used to produce the 297 × 17 datasets. (**C**) Dimension one of multidimensional scaling (MDS) plots of one-to-one Robinson Foulds distances of 297 trees produced from amino acid and nucleotide sequences.

The presence of short backbone branches leading to *Crocodia*, *Parmostictina*, *Podostictina*, *Pseudocyphellaria*, *Sticta*, and *Yarrumia* is suggestive of a rapid diversification (Fig. 2). To investigate the cause for inconsistent phylogenetic tree results based on potential rapid diversification and gene-tree discordance, we created a pruned dataset (Fig. 3) (297 × 17). This created a complete matrix without missing data which was analyzed with RAxML and ASTRAL based on nuclear data and their amino acid translations. All four trees differed in their backbone branching patterns (Fig. 3A), with incongruent clades receiving strong support. Removing *Podostictina* and *Yarrumia* from the datasets, especially *Podostictina*, increased congruence and node support among all trimmed datasets (Supplementary Fig. S1). To find the most common topology we used SumTrees, which recovered 297 unique topologies of each tree reconstruction from the pruned nucleotide and amino acid datasets. We visualized this gene-tree discordance by using DensiTree, which creates a figure by overlaying all 297 gene trees. Although both datasets recovered clear groupings representing well supported clades, the backbone is depicted as a diffuse cloud of branches (Fig. 3B). Using the pairwise Robinson-Foulds distances (topological distances) of gene-trees and multidimensional scaling (MDS), we found that clustering of topologies was not significant and that both the amino acid and nucleotide datasets exhibited a diffuse pattern in all dimensions (Fig. 3C). This suggests that the gene-trees in both amino acid and nucleotide datasets have no clear pattern of a particular topology.

**Figure 4:**
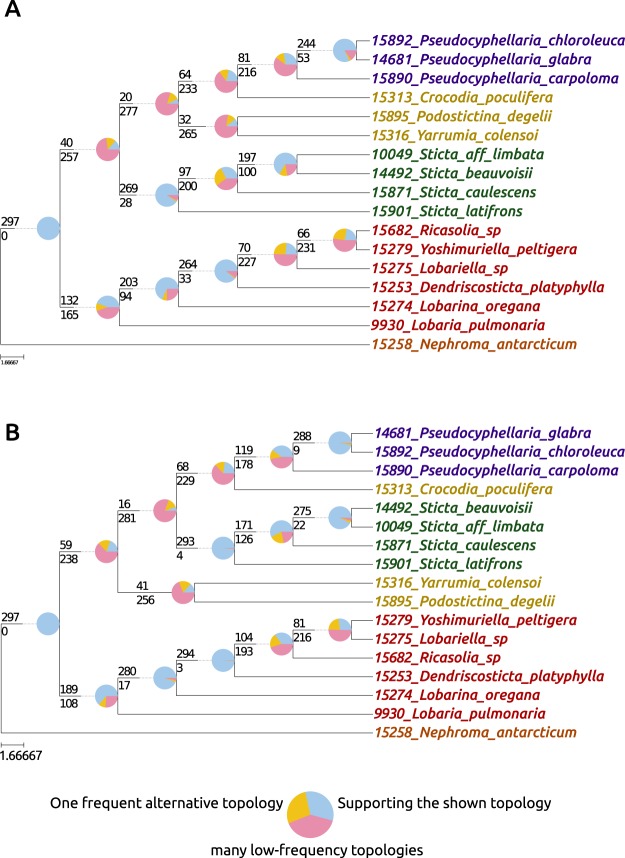
ASTRAL species trees. (**A**) Estimated with the 297 amino acid trees. (**B**) Estimated with 297 nucleotide trees. Pie charts show the gene tree conflict evaluation at each node with light blue proportions representing concordant topologies, pink portions representing conflicting topologies, and yellow proportions representing one dominant alternative topology. Numbers above the branches report the number of gene trees supporting that node, while the number below reports the number that are in conflict.

We also used the program PhyParts to visualize the amount of gene trees that were in support or conflict with each node in the 297 × 17 aa and 297 × 17 nuc datasets (Fig. 4). ASTRAL species trees generated from the gene trees served as reference trees. The proportion of gene trees that supported and conflicted with reference bipartitions were mapped to the reference trees. The PhyParts output for both datasets showed that the deep backbone nodes leading to the genera *Crocodia*, *Podostictina*, *Pseudocyphellaria*, *Sticta*, and *Yarrumia* were supported by a small plurality of gene trees (Fig. 4, pink portions of the pie charts). The node reprsenting the common ancestor of *Crocodia*, *Podostictina*, *Pseudocyphellaria*, *Sticta*, and *Yarrumia* was only supported by 13% and 20% of the gene trees in the amino acid (Fig. 4A) and nucleotide (Fig. 4B) datasets respectively. However, some nodes within this clade were congruent among a vast majority of amino acid and nucleotide gene trees, such as the stem node leading to the four *Sticta* species and the node leading to *P*. *chloroleuca* and *P*. *glabra*. The deeper reference tree node leading to *Dendriscosticta*, *Lobaria*, *Lobariella*, *Lobarina*, *Ricasolia*, and *Yoshimuriella* was supported by 44% and 63% of the amino acid and nucleotide gene trees respectively. The more recent nodes representing the relationships of *Lobariella*, *Ricasolia*, and *Yoshmuriella* also conflicted with amino acid and nucleotide gene trees, but the remaining nodes in this clade were generally more supported.

### Evidence for reticulate evolution in Lobariaceae

**Figure 5:**
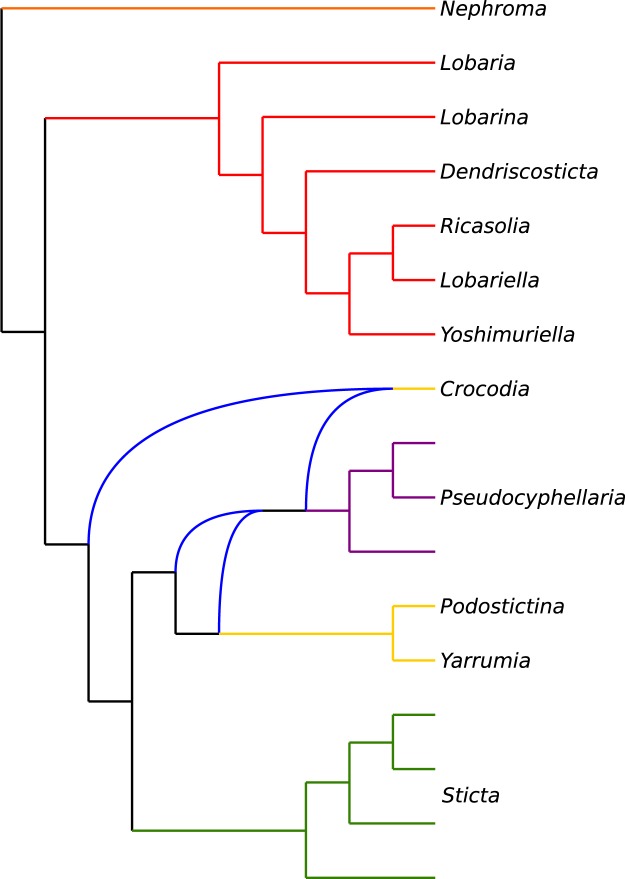
PhyloNet network showing one of the most likely reticulation scenarios.

We used the same pruned 297 × 17 nuc dataset to infer evidence of reticulate evolution in Lobariaceae by using PhyloNet. Although, the maximum pseudo-likelihood (MPL) option (InferNetwork_MPL) in PhyloNet indicated that a three-reticulation model had the best fit to our data, none of the networks produced by PhyloNet had three reticulations (Table 2). At most, two reticulations were inferred (Fig. 5). The reticulations reconstructed in the networks generally originated in the ancestral nodes or branches of samples from *Crocodia*, *Podostictina*, *Pseudocyphellaria*, *Sticta* and *Yarrumia*. Using a full likelihood approach (InferNetwork_ML) on 17 taxa is too computationally heavy for most servers hence we produced a dataset that had only the five taxa that were most involved in putatively reticulate patterns in the 17-tip analyses (*Crocodia*, *Podostictina*, *Pseudocyphellaria*, *Sticta*, and *Yarrumia*). As with the MPL approach, the three-reticulation scenario had the best fit to the data in the ML analysis and the most likely network had three reticulations (Table 3 and Supplementary Figs S2 and S3).

**Table 2:** Total log probabilities and AIC scores for different reticulation scenarios using the MPL approach.

Reticulations	k	Total log probability	AIC	
0	**32**	−4,772.61	9,609.22	
		−4,772.93	9,545.87	
		−4,773.10	9,546.21	
		−4,774.06	9,548.11	
		−4,774.08	9,548.16	
		−**4**,**773**.**36**	**9**,**546**.**71**	Average
1	**33**	−4,757.90	9,581.80	
		−4,766.82	9,533.64	
		−4,770.67	9,541.33	
		−4,773.04	9,546.08	
		−4,774.05	9,548.10	
		−**4**,**768**.**50**	**9**,**536**.**99**	Average
2	**34**	−4,749.11	9,566.22	
		−4,771.13	9,542.27	
		−4,771.34	9,542.68	
		−4,772.93	9,545.87	
		−4,774.05	9,548.11	
		−**4**,**767**.**71**	**9**,**535**.**43**	Average
3	**35**	−4,687.22	9,444.44	
		−4,743.86	9,487.72	
		−4,760.41	9,520.82	
		−4,772.93	9,545.87	
		−4,773.04	9,546.07	
		−**4**,**747**.**49**	**9**,**494**.**98**	Average
4	**36**	−4,769.16	9,610.31	
		−4,770.96	9,541.91	
		−4,771.03	9,542.07	
		−4,771.78	9,543.57	
		−4,772.94	9,545.87	
		−**4**,**771**.**17**	**9**,**542**.**35**	Average
5	**37**	−4,770.67	9,615.33	
		−4,772.11	9,544.21	
		−4,773.10	9,546.21	
		−4,774.05	9,548.11	
		−4,774.07	9,548.15	
		−**4**,**772**.**80**	**9**,**545**.**60**	Average
10	**42**	−4,770.66	9,625.33	
		−4,770.96	9,541.92	
		−4,772.93	9,545.87	
		−4,773.04	9,546.07	
		−4,773.32	9,546.63	
		−**4**,**772**.**18**	**9**,**544**.**36**	Average

**Table 3:** Likelihood and AIC scores for different reticulation scenarios using the ML approach.

Reticulations	k	Total log probability	AIC
0	7	−1435.24	2870.48
1	8	−1366.30	2734.60
2	9	−1335.74	2675.48
3	10	−1328.20	2662.40

**Figure 6:**
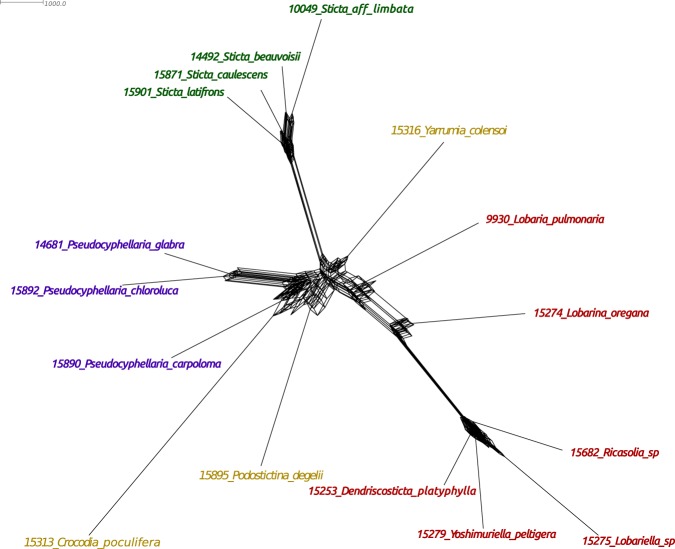
A SplitsTree network estimated with the MedianNetwork method. The network shows relationships of genera based on the 297 × 17 nuc dataset with no outgroup.

To further investigate the evidence for reticulations, we used the MedianNetwork analysis implemented in the program SplitsTree. Instead of gene trees for input, which is what is used in Phylonet, we used the concatenated 297 × 17 nuc alignment. We removed the outgroup *Nephroma antarcticum* for the network analysis. The SplitsTree MedianNetwork analysis recovered the well-supported and differentiated ML and ASTRAL lineages such as the *Sticta*, *Pseudocyphellaria* and the clade with *Dendriscosticta*, *Lobariella*, *Ricasolia*, and *Yoshmuriella*, but also showed numerous box-like relationships indicating conflicting phylogenetic signals (Fig. 6).

### Timing of divergence in Lobariaceae

**Figure 7:**
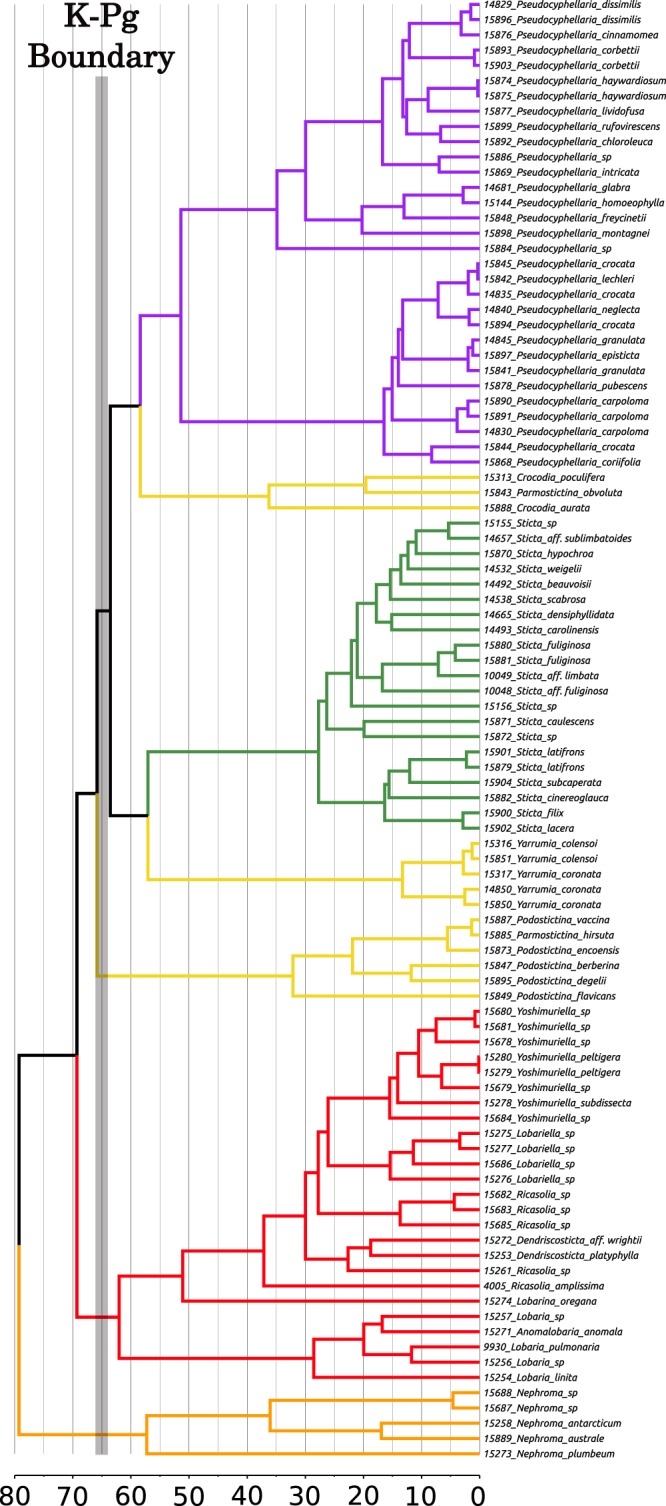
A chronogram estimated by Bayesian relaxed molecular clock implemented in MCMCTree. The ML tree inferred with concatenated amino acid sequences (376 × 96 aa - congruent with the ASTRAL species tree) was used a scaffold for the dating analysis. The grey vertical bar depicts the K-Pg boundary. The timescale is in units of millions of years. Colors of clades: Orange: *Nephroma* (outgroup); Red: *Anomolobaria*, *Dendriscosticta*, *Lobaria*, *Lobariella*, *Lobarina*, *Ricasolia*, *Yoshimuriella*; Yellow: *Crocodia*, *Parmostictina*, *Podostictina*, and *Yarrumia* (not monophyletic); Green: *Sticta*; Purple: *Pseudocyphellaria*.

Both runs of MCMCTree converged on similar estimations (Table 4). The average stem age of Lobariaceae was estimated to be 64.4 Mya (91.9–43.4 Mya). Crown divergences of the major clades in Lobariaceae are as follows: *Lobaria s*.*lat* clade (which includes *Anomolobaria*, *Dendriscosticta*, *Lobariella*, *Lobarina*, *Ricasolia*, and *Yoshimuriella*), 57.6 Mya (84.0–38.1 Mya); *Podostictina*, 29.5 Mya (53.0–14.2 Mya); *Yarrumia*, 12.4 Mya (21.2-5.9 Mya); *Sticta*, 25.2 Mya (39.9–15.2 Mya); *Crocodia*, 33.4 Mya (56.0–16.7 Mya); and *Pseudocyphellaria*, 54.1 Mya (78.7–35.2 Mya). The estimates of the first run are depicted in Fig. 7.

**Table 4:** Node ages estimated in MCMCTree with error ranges in millions of years.

Clades	Run 1			Run 2			Averaged runs		
	age	minimum	max	age	minimum	max	age	minimum	max
Lobariaceae	***69.3***	53.4	89	***59.4***	33.4	94.7	***64.4***	43.4	91.9
*Nephroma*	***57.3***	36.6	86	***53.9***	31.2	84.6	***55.6***	33.9	85.3
*Lobaria* s.lat clade	***62***	46.7	81.6	***53.2***	29.5	86.4	***57.6***	38.1	84.0
*Lobaria*	***28.5***	20	42.5	***25.2***	14.7	41.1	***26.9***	17.4	41.8
*Dendriscosticta*	***22.7***	13.9	35.2	***19.2***	9.6	37.4	***21.0***	11.8	36.3
*Ricasolia*	***13.7***	7.7	21.1	***11.4***	5	20.4	***12.6***	6.4	20.8
*Lobariella*	***15.4***	9.4	22.2	***12.9***	6.1	21.8	***14.2***	7.8	22.0
*Yoshimuriella*	***15.5***	10.2	21.6	***13.1***	6.6	21.7	***14.3***	8.4	21.7
*Podostictina*	***32.1***	16.8	54.6	***26.8***	11.5	51.3	***29.5***	14.2	53.0
*Yarrumia*	***13.3***	6.8	21.7	***11.5***	5	20.6	***12.4***	5.9	21.2
*Sticta*	***27.7***	18.5	43.2	***22.6***	11.8	36.6	***25.2***	15.2	39.9
*Crocodia*	***36.2***	20	56.3	***30.6***	13.4	55.6	***33.4***	16.7	56.0
*Pseudocyphellaria*	***58.4***	43.2	76.2	***49.8***	27.2	81.1	***54.1***	35.2	78.7

## Discussion

We produced the first target enrichment dataset of a lineage of lichenized fungi with a goal of resolving the higher-level relationships in Lobariaceae, and found that the evolutionary history exhibited a rapid and reticulate diversification and that we could not confidently reconstruct the relationships assuming a strictly bifurcating tree despite the wide sampling of nuclear loci. The phylogenetic positions of certain lineages differ when using (1) different data types (nucleotides versus amino acids) or (2) different tree reconstruction methods (Fig. 1). However, more slowly-evolving amino acid sequence data yielded more consistent results, especially with the 376-locus dataset, where the concatenated RAxML and ASTRAL species tree backbone branching patterns were identical (Fig. 2). This topology was more supported in ASTRAL because the node leading to the *Podostictina* clade in the RAxML tree was poorly supported (Fig. 1B). An increasing number of studies are producing datasets of hundreds, even thousands of loci, and are finding that certain nodes in the tree of life are still very challenging to resolve with our current inference methods^4–8,11–13^. We found no exception in Lobariaceae. However, even though phylogenetic relationships among genera remained uncertain (c.f., Moncada, Lücking, and Betancourt-Macuase 2013), we were able to further study the factors that are preventing resolution. As a result, our study is the first on lichenized fungi to investigate with target capture, the underlying mechanisms that may lead to poorly-resolved and conflicting relationships.

Regardless of the dataset or analysis, backbone branches marking shared ancestry of *Pseudocyphellaria* (including *Crocodia*, *Podostictina*, and *Yarrumia*) and *Sticta* were very short, suggesting a rapid diversification. Short branches can be difficult to resolve with confidence, even with large subgenomic datasets^40^. Rapid diversifications can also lead to conflicting topologies among individual gene trees^15^. This is consistent with our study, even in our reduced dataset (297 × 17) with 297 unique rooted topologies (Figs 3 and 4). The multi-species coalescent-based species tree approach can account for ILS^41^. However, species trees with short, deep branches, such as those recovered in this study, can result in uninformative gene-trees, and the gene trees may result in erroneous species tree reconstruction^42^. The inconsistency in phylogenetic reconstruction and low support for certain branching patterns revealed in our study suggest that the short branches in our trees confound species tree approaches in this case. The only time we found a consensus between the two methods was when we used the largest amino acid data set (376 × 96 aa), but node support was low for some of these relationships (Figs 1B and 2).

Concatenation of genetic loci has a long history in phylogenetics, but it has also been known to produce highly supported yet conflicting results, especially in the area of tree space known as the “anomaly zone”^43^ (when most gene trees in the genome do not reflect the true speciation history). Furthermore, concatenated analyses cannot account for the different evolutionary histories at different loci, which can also produce misleading topologies^44^. They can also be extremely sensitive to outlier loci^45^. The probability of sampling an outlier locus increases as the amount of data increases, and it is therefore expected that the effect of such loci will be stronger in larger subgenomic data set^45^.

Models that incorporated reticulations always had better fit to the data than models assuming a strictly bifurcating tree (Fig. 5, Tables 2 and 3). Strictly bifurcating trees are optimized and reduced visualizations of a more complicated biological reality and when using hundreds of genes, a network will generally be a better explanation of the data^46^. In most of the phylogenetic networks generated, the taxa with labile topological positions (*Podostictina*, *Sticta* and *Yarrumia*) were usually associated with the reticulations in the network. The Phylonet reticulation estimations were supported by the PhyParts analysis (Fig. 4), which showed that most of the gene trees were in conflict with the ASTRAL species trees at the deeper nodes connecting to *Crocodia*, *Podostictina*, *Pseudocyphellaria*, *Sticta* and *Yarrumia*, the genera associated with reticulations. Furthermore, the SplitsTree network (Fig. 6) showed that most of the nodes associated with the clade containing genera with pored lower cortices contain relatively more reticulations than the *Lobaria* s.lat. clade and that the phylogenetic placement of *Podostictina* and *Yarrumia* is unclear, as they originate from the base of the network. Reticulation events can confound both concatenated ML and coalescent-based species tree approaches, causing incongruence and poorly supported relationships^14,20^. This could also contribute to the inconsistency among phylogenetic reconstructions and poor nodal supports found in our study. Additionally, the effects of reticulation and rapid diversification may not be independent. Reticulations have been hypothesized to promote subsequent rapid diversification events in cichlids^47^, yeasts^48^, and fungal pathogens^49^. Reticulate evolution is most likely to induce rapid diversification when parental lineages occupy highly dissimilar ecological niches but are only moderately genetically differentiated^50^. Recombination among distinct yet compatible evolutionary lineages may generate novel phenotypes to exploit new niches. Although we have shown that reticulate models have a better fit, it is unclear whether they led to a rapid diversification (suggested by the short and poorly supported nodes) in Lobariaceae as the two are not mutually exclusive. Rapid radiations could also increase the chances of reticulate evolution, because so many closely related taxa may not have had the chance to develop effective reproductive barriers before secondary contact occurred in nature. Reticulate evolution may characterize the evolution of a number of lichenized fungi. Hybridization has been invoked to account for patterns in secondary chemistry in *Hypotrachyna*^51^ and evidence for introgressive hybridization was proposed in the *Peltigera didactyla* complex^52^. Also gene-tree incongruence in *Cladonia*^53^ and *Lobaria*^54^ were seen as compatible with reticulation. In *Letharia* a reticulate evolutionary history was proposed based on patterns of recombination in nuclear DNA markers^55^. The other form of reticulate evolution, horizontal gene transfer (HGT), has been discovered with fungal genes being found in the *Trebouxia* photobionts^56^, and with polyketide synthases of actinobacterial origin being found in lichenized fungi^57^, but HGT has not yet been reported among lichenized fungi and is not hypothesized in this study.

Our topology (376 × 96 aa data sets in Figs 1B and 2) was similar to that of Moncada *et al*.^27^ with the main exceptions being the relationships of *Sticta* and genera previously segregated from *Pseudocyphellaria* (e.g. *Crocodia*, *Parmostictina*, *Podostictina*, and *Yarrumia*). In the previous study^27^, *Parmostictina*, *Podostictina* and *Crocodia* formed a well-supported clade (*Yarrumia* was not included in their analysis) sister to *Pseudocyphellaria*. In our analyses, the *Crocodia* and *Pseudocyphellaria* still formed a sister-group relationship in all datasets, but the relationships of *Parmostictina* and *Podostictina* were poorly supported and differed depending on data type and method of inference: it could be placed sister to either all Lobariaceae, to (*Crocodia* + *Pseudocyphellaria* + *Sticta* + *Yarrumia*), to *Yarrumia*, or only to *Sticta* (Figs 1, 2, and 3). The erratic placement of *Parmostictina* and *Podostictina*, when using different data types and inference methods, exhibits patterns found in other unrelated groups of organisms with unresolved phylogenetic relationships, such as the deep, poorly supported nodes leading to ctenophores and sponges^4,5^, *Amborella*^9–12^, and Gnetales^13,58^. Another relationship that was often but not always recovered was the *Sticta* and *Yarrumia* sister relationship.

Our time-calibrated phylogeny (Fig. 7) recovered a nearly 70 Mya stem age of Lobariaceae which is in agreement with the estimate of Gaya *et al*.^35^ but not with Simon *et al*.^36^ where a 50 Mya stem age was reported. Furthermore, our crown age estimates agree with other dated phylogenies generated for the genera *Sticta*^38^ and *Lobaria*^37^, both of which have crown ages around 30 Mya. The timing of the rapid and reticulate Lobariaceae divergence is near the end of the Cretaceous around 70–60 Mya. The Cretaceous Terrestrial Revolution^59,60^ (CTR) occurred between 100 and 70 Mya and is associated with the massive diversification events that gave rise to the angiosperms^61,62^. The increase in angiosperm diversity in the late Cretaceous created new ecological opportunities^63,64^ and these may have triggered bursts of diversification in spore-dispersing plants^63–66^ mammals^67^, and ants^68^. Secondary diversification pulses following the CTR and rise of angiosperms are especially linked to the evolution of epiphytism and has been demonstrated in leafy liverworts^65^ and ferns^63,64^. Most Lobariaceae species are epiphytic and the rapid diversification event seen in our study could be another example of a subsequent pulse in divergence linked to the CTR. The rapid diversification of Lobariaceae was also associated with the Cretaceous–Paleogene (K-Pg) boundary around 66 Mya. Mass extinctions associated with K-Pg boundary may have reduced competition and provided an opportunity for surviving lineages to radiate^69^. The macrolichen growth form is associated with multiple diversification rate increases following the K-Pg boundary (Huang *et al*. 2019 unpublished data^70^). Lobariaceae, a lineage composed solely of macrolichens, may have also experienced a similar diversification rate increase as in other clades of lichenized fungi.

Incongruence in phylogeny reconstruction can be caused by stochastic and systematic errors. Stochastic error is exacerbated by gene length. The shorter the sequence, the more stochastic error can cause misleading results. With hundreds of genes, phylogenomic approaches, such as target-capturing applied in this study alleviate the issue of stochastic error, but systematic errors may be amplified when phylogenetic reconstruction methods do not account for the properties in the data^14,71^. Overestimation of divergence times has been demonstrated when molecular substitution rates vary among clades and when only a subset of taxa are used in the phylogeny reconstruction^72^. While it is unclear whether our dataset is characterized by clade-specific rate heterogeneity, our taxon sampling is not complete. This incomplete sampling could lead to an over estimation of divergence times in our dataset and shift the occurrence of the rapid diversification event after the K-Pg boundary. Furthermore the presence of short branches in our phylogenetic reconstructions and a high level of gene tree discordance could be caused by ILS and reticulate evolution. It is possible that these historical processes also influenced divergence time estimations as well. If ILS is present at certain loci, divergence times will be overestimated with these genes because coalescence of the loci precedes divergence of species^73^. Hybridization could have multiple effects on divergence time estimates depending on whether the hybrid locus coalesced before or after species divergence. Hybrid loci that coalesce before species divergence would lead to overestimated divergence times similar to ILS. However, if hybrid loci coalesce after lineage splitting, divergence times will be underestimated^73^. Because we did not identify which loci were influenced by ILS and hybridization, it is important to state that the divergence times estimated in this study should be interpreted with caution.

Since the application of genetic data to reconstructing the evolutionary relationships of lichenized fungi, our understanding of the diversification of most lineages has significantly improved. Single and multiple locus phylogenies have discovered many relationships that were obscured by homoplasy in phenotypic characters. With the advent of next-generation sequencing (NGS) technology, only a handful of studies have been conducted on groups of lichenized fungi to resolve phylogenetic relationships^74,75^. In traditional phylogenetic studies, fungal-specific primers got around the issue of mixed genomes in DNA isolations, but with genomic studies, the only way to get purely fungal DNA is to culture the fungal symbiont. The ability of lichenized fungi to be cultured varies among groups and it has not been successful in many. Regardless, over ten genomes have been sequenced and are available for study. Recently, studies using purely fungal reference genomes from cultured lichenized fungi have gotten around the issue of using metagenomic DNA isolations, by applying a mapping step that pulls out the fungal reads for use in phylogenomic analyses^75^.

This study is the first to apply the target capturing approach in lichenized fungi to investigate historical processes influencing the evolution of major clades in Lobariaceae. Although we were not able to confidently resolve certain relationships, we were able to investigate why certain nodes remained unresolved, and we have a more thorough understanding of the evolutionary history of the group. We provide evidence for rapid diversification and reticulate evolution in our data set, which would not be possible to infer with only a few loci. However, it is still not clear what the relative effects of rapid diversification and reticulations are on gene tree discordance and difficulties in species tree reconstruction. We encourage the phylogenetic study of taxonomic groups with uncertain phylogenetic relationships so, as a scientific community, we can understand how historical processes are most likely to obscure relationships in the tree of life. Resolution of some nodes in the tree of life may not always be possible with increasing datasets, but a detailed understanding of the evolutionary events that confound the history of a group are the next best steps to take to make informed decisions about their taxonomic classification.

## Methods

### Taxon sampling

We sampled representatives of all the current and tentative genera in Lobariaceae. Our dataset includes representatives of the three major classic genera *Lobaria*, *Pseudocyphellaria*, *Sticta*, along with representative samples of the later segregated genera (*Anomolobaria*, *Crocodia*, *Dendriscosticta*, *Lobariella*, *Lobarina*, *Parmostictina*, *Podostictina*, *Ricasolia*, *Yarrumia*, and *Yoshimuriella* (Supplementary Table S2).

### Bait design

We designed baits for target capture using Markerminer^76^, a bioinformatics pipeline that finds single copy loci in the genome using genome and transcriptome data as inputs. Markerminer is designed for use with angiosperms, and has databases for 15 angiosperm genomes, but other genome databases can be added for customized use of the program. We used the *Lobaria pulmonaria* genome and the gene annotation file (Lobpul1_AssemblyScaffolds_Repeatmasked.fasta and Lobpul1_GeneCatalog_20170213.gff3) from JGI (https://jgi.doe.gov/) to develop a custom database. The result is a simplified reference genome that only contains gene regions and the introns are hard-masked as “N”s in the sequence^77^.

We assembled transcriptome data (published in Meiser *et al*.^77^) from *Evernia prunastri*, *Pseudevernia furfuracea* and *Lasallia pustulata* using Trinity^78^. The resulting transcriptomes and the custom *L*. *pulmonaria* database were used to identify clusters of single-copy gene transcripts present in the transcriptome assemblies. Next, these were aligned and filtered against the *L*. *pulmonaria* reference proteome from JGI (Lobpul1_GeneCatalog_proteins_20170213.aa.fasta) using BLAST. These single-copy genes were re-aligned to the intron hard-masked genome and intron-exon boundaries were identified.

Markerminer identified and aligned 1,714 single-copy genes to the hard-masked *L*. *pulmonaria* reference genome. We selected loci that had at least one exon of at least 500 bp and indicated clear intron-exon boundaries on the hard-masked alignment. The designed baits were collected as 800 separate fasta DNA sequence files from the *L*. *pulmonaria* and *E*. *prunastri* (400 each). These sequences were provided to Arbor Biosciences (Ann Arbor, MI, USA) for MYbaits bait design. These filtered baits covered 92% of the desired target positions with at least one bait, therefore all 800 target sequences are represented with at least one bait. One hundred nucleotide-long baits were designed with two times tiling density resulting in 18,139 raw unfiltered baits. Following Arbor Biosciences recommended filtering process (baits passing “Moderate” BLAST filtering), 17,941 baits were retained for target-capturing.

### Library preparation

DNA was obtained using the ZR Fungal/Bacterial DNA MiniPrep™ (Zymo Research, Irvine, CA, USA) or by a CTAB extraction protocol. The concentration of all DNA isolates was quantified with the Qubit (Thermo Fisher Scientific, Waltham, MA, USA). Two hundred ng of meta-genomic DNA was normalized to a final volume of 52.5 μL in resuspension buffer. Fragments of DNA around 500 bp were generated with M220 Focused-ultrasonicator™ (Woburn, MA, USA) and then 50 μL was cleaned up with 80 μL SeraPure beads which are an inexpensive alternative to commercially purchased magnetic beads^79^. The Adapterama dual-indexing system^79^ was used to uniquely barcode all samples using the KAPA Hyper Prep Kit (KAPABiosystems, Wilmington, MA, USA). Twenty-five μL of the sheared, cleaned bead elution was used in a 30 μL end-repair and A-tailing reaction followed by a 55 μL ligation to attach the Adapterama stubby y-yolk adapter. The ligation products were subjected to bead-based size selection with SeraPure bead to enrich for fragments of around 550 bp which was eluted in 22 μL of RSB. Twenty μL of the elution was used in a limited-cycle (9–11 cycles) polymerase chain reaction (PCR) to attach the barcoded iTru5 and iTru7 Adapterama primers. Subsequently, the PCR products were cleaned with 1X SeraPure beads and eluted in 43 μL of nuclease-free, PCR-grade water. DNA concentrations of all samples were quantified with a Qubit fluorometer (Thermo Fisher Scientific, Waltham, MA, USA) and a subset were checked for proper size distribution of fragments on the Bioanalyzer (Agilent, Santa Clara, CA, USA).

Samples were pooled by phylogenetic relatedness, corresponding to major clades (corresponding to the colors in Figs 1, 2, and 3) of the previous Lobariaceae three-locus phylogeny^27^, for hybridization with RNA baits. For each sample, 100–200 ng of DNA was mixed with all samples of each of the five pools and then concentrated in a heated vacuum centrifuge. Pools were hybridized with reagents provided with the baits from Arbor Biosciences for ~20 h at 65 °C. After incubation, the baits were attached to Dynabeads® M-280 Streptavidin beads (Carlsbad, CA) and then washed according to the Arbor Biosciences protocol followed by a post-wash enrichment PCR cycle with KAPA Hifi Hotstart ReadyMix. Each pool went through 11 cycles of PCR except for the outgroup (*Nephroma* samples), which was subjected to 14 cycles. These were cleaned with 1X SeraPure beads and the DNA concentration was quantified on the Qubit and size of distribution of the DNA fragments in the pools were observed on the Bioanalyzer. These pools were mixed together to have 3 ng of DNA per sample in the final 96-sample pool which was used for sequencing at the Field Museum’s Pritzker Laboratory with a single 300-cycle v2 MiSeq reagent kit (Illumina, San Diego, CA, USA).

### Data processing

The MiSeq run (300 cycles using V2 chemistry) produced 15,835,491 150-bp paired-end reads, which were demultiplexed and adapter-trimmed by Illumina BaseSpace. The raw reads were downloaded to the Field Museum server and quality trimmed with Trimmomatic^80^ using a quality cut off of 15 in a 4-bp sliding window, discarding any reads under 35 bp. Only paired, trimmed reads were used in downstream analyses, which is an average of 158,443 reads per sample remaining after trimming with a range from 7929 to 317,874 reads (Supplementary Table S1). These sequences were used for the read files using the program HybPiper^39^ which assembles gene regions and extracts exon sequences for each sample. We generated a target file from the *L*. *pulmonaria* transcriptome on JGI (Lobpul1_GeneCatalog_proteins_20170213.aa.fasta) that has the complete amino acid sequences of each of the 400 target genes that were used for bait design. The sorted, trimmed reads of each sample were mapped to the targets using the default BLASTX^81^ option, which we found recovered much more data (a more complete dataset), especially for the outgroup (*Nephroma* taxa) than the BWA option that uses a nucleotide target file. Upon completion of the HybPiper assembly, the 399 gene sequences (one gene did not produce any sequences) were extracted using retrieve_sequences.py and then batch aligned using MAFFT^82^.

HybPiper will flag a gene if it identifies multiple sequences spanning at least 85% of the gene length^39^. When this occurs at a specific gene, HybPiper will choose only one of the sequences, first by selecting the sequence with the highest depth of sequencing and then, if all sequences are similar in coverage, it uses the closest match to the specified target sequence file. For the paralog warning genes, all of the copies were extracted (paralog_retriever.py), aligned and phylogenetically analyzed to check the patterns of paralogy for each of these flagged genes. However, paralogous sequences could be present in some datasets that did not have a warning. For example, if one or more taxa yield only one sequence that is paralogous to the others. Furthermore, multiple copies could be alleles or a duplication that postdates a bifurcation which HybPiper would flag, but this case would not cause problems with phylogenetic inference. Future studies will need to use more thorough analyses to have a detailed understanding of how paralogs are influencing phylogenetic reconstructions.

### Concatenation of datasets

Multiple nucleotide alignments of single genes were concatenated with FASconCAT-G^83^. One alignment contained 376 loci and 96 tips (376 × 96 nuc) and had 438,036 nucleotide positions with 13.75% gaps and undetermined characters. Only genes that had at least 48 sequences (50% sample representation) were used. Another reduced dataset (297 × 17 nuc) with only 17 taxa, representing all Lobariaceae genera and the well supported subclades in *Sticta* and *Pseudocyphellaria* was generated in HybPiper. With this dataset 297 of the genes had all 17 sequences and these alignments were used to generated gene trees and a concatenated alignment with 338,019 nucleotide positions and 4.04% gaps and undetermined characters. We also generated the same concatenated datasets with amino acid sequences using FASconCAT-G. The dataset with 376 loci and 96 tips (376 × 96 aa) and had 145,467 amino acid positions with 6.34% missing data and 7.26% indels. Finally, the reduced dataset (297 × 17 aa) of 297 loci and 17 tips had 112,265 amino acid positions with no missing data, but it did contain 3.89% indels. A summary of all datasets can be found in Table 1.

### Maximum likelihood and species tree analyses

For all full and reduced datasets, concatenated phylogenies and all single gene trees were estimated using the RAxML^84^ rapid hill climbing algorithm and performing 100 bootstrap replicates. All single gene alignments and all partitions of each concatenated dataset had substitution model selection using either the GTRGAMMA or PROTGAMMA -AUTO models for nucleotide and amino acid models of sequences respectively. Since concatenated datasets can be prone to false positive topologies^43,85^, we also analyzed our datasets with the multi-species coalescent model the program Accurate Species Tree Algorithm (ASTRAL 5.6.1)^86^. For the ASTRAL analyses, we inferred species trees from single, fully resolved, and unrooted gene trees mentioned above. Branch support was reported as local posterior probabilities (i.e., the support for a quadripartition).

### Analysis of gene-tree discordance and tree space

Regardless of the data type or tree building approach, all of the datasets produced backbones with short internal branches (Fig. 3). During rapid diversification, speciation and gene flow occur simultaneously in a relatively short period of time, which can lead to high levels of gene-tree discordance. Discordance among gene-trees can be the result of incomplete lineage sorting (ILS), reticulate evolution, and homoplasy. The coalescent model implemented in ASTRAL, accounts for ILS, but this approach was still unable to completely resolve the deep divergences in the backbone of the amino acid and nucleotide datasets. Although this does not completely rule out ILS as a source of gene-tree discordance, it does suggest that other historical processes, such as homoplasy or reticulate evolution, which are not accounted for in ASTRAL, were potentially contributing to the poorly resolved backbones in our reconstructions. To conduct analyses on and visualize the gene-tree discordance the best trees from the RAxML analyses for each gene were converted to ultrametric trees using the chronos() function in the R package APE (Paradis, Claude, and Strimmer 2004) with a lambda = 1. This was done using the program SumTrees^87^ implemented in DendroPy 4.0.0^88^ to find if there was a common tree topology among the reduced (297 × 17) amino acid and nucleotide datasets. DensiTree 2.2.5^89^ was used to visualize the discordance of gene-tree topologies.

Tree space of the reduced datasets (297 × 17 nuc and 297 × 17 aa) were described using multidimensional scaling (MDS) in R using the customized topclustMDS function described elsewhere. These datasets were used because complete trees (all having 17 tips) were required. Gene trees were loaded into R using the APE package^90^, then MDS scaling and functions in the cluster package^91^ were used to identify the most appropriate number of clusters and the identity of loci comprising each cluster.

We used the program PhyParts^92^ (https://bitbucket.org/blackrim/phyparts) to assess the level of concordance and conflict among gene trees in the reduced datasets (297 × 17 aa and 297 × 17 nuc). The ASTRAL species trees generated from each dataset were used as reference trees. Individual amino acid and nucleotide gene trees were rooted with the root.multiphylo() function in R package APE with *N*. *antarcticum* as outgroup. PhyParts conducts a bipartition analysis across all rooted gene trees and maps the amount of trees that support and conflict with each bipartition in the reference tree. The output of PhyParts was visualized by plotting pie charts that depict the amount of gene trees that supported and conflicted with each bipartition on the ASTRAL species tree with the script phypartspiecharts.py (https://github.com/mossmatters/MJPythonNotebooks).

### Tests for reticulate evolution

Another known source of poor backbone node support in phylogenies is reticulate evolution. This process is not modeled in the most used phylogenomic inference programs such as RAxML or ASTRAL as the output can only be bifurcating. A current program, PhyloNet^93^, does model this process, and we used it to investigate whether parts of the relationships in the Lobariaceae phylogeny are better supported with a model of reticulate evolution rather than a strictly bifurcating tree. PhyloNet identifies reticulation events using a multi-species network coalescent model that accounts for ILS and reticulate evolution. We first used the maximum pseudo-likelihood approach (InferNetwork_MPL option) on the 297 × 17 nuc dataset, specifying the -po option which optimizes the branch lengths and inheritance probabilities under full likelihood for the returned species networks. We conducted six analyses with reticulation scenarios ranging from zero to five and then ten with each analysis conducting ten runs and inferring five networks. The zero-reticulation scenario served as a null model and we then increased the reticulation events step-wise to five reticulations to see how each increase fit to the data by calculating AIC for each network. Finally, we conducted a ten-reticulation analysis to see if more than five reticulations could be recovered from our dataset.

Although the MPL approach is computationally fast, under certain conditions, the true reticulation history may not be identifiable^94^. Thus, using the drop.tip() function in the R package APE^90^, we produce a five-tip dataset that contain only *Crocodia*, *Podostictina*, *Pseudocyphellaria*, *Sticta*, and *Yarrumia (*the genera that were usually involved in the reticulations of the MPL analyses), so that we could apply a fully parameterized ML approach (InferNetwork_ML). Due to the taxing computational demands of the fully parameterized ML option of PhyloNet, we only tested zero to three reticulations (using the -po option described above), because the best fitting scenarios in the MPL analyses included three reticulations (see results). Each analysis conducted five runs and produced one network and the total log probabilities were used to calculate AIC to assess model fit.

To visualize uncertainty and phylogenetic conflict within our reduced dataset, we used SplitsTree v4.14.8^95^. The input was the concatenated reduced nucleotide dataset (297 × 17 nuc) and excluded the outgroup *N*. *antarcticum* since evidence for reticulate evolution was only detected among the ingroup taxa in Phylonet. The data were analyzed using the MedianNetwork method which uses all sites in the nucleotide alignment that contain exactly two different states while excluding gaps or missing states to generate an unreduced median network.

### Dated phylogeny of Lobariaceae

The program MCMCTree 1.2^96,97^ was used to infer divergence times in Lobariaceae. The ML tree (376 × 96 aa), inferred with amino acids and congruent with the one inferred in ASTRAL (Fig. 2A) was used for the dating analyses and the concatenated amino acid matrix. We used CODEML to generate the Hessian matrix using an empirical rate matrix with gamma rates among sites (WAG + Gamma). Using this matrix, we ran MCMCTree with appropriate rates under the approximate method. Settings were set as follows: independent-rates model, time unit at 100 Ma, sampling fraction at ρ = 0.1, and the birth and death rates at λ = µ = 1. We ran the analyses with gaps and ambiguities removed (cleandata = 0). The gamma prior for the substitution model was set at six transitions to two transversions rate ratio. The gamma shape parameter for variable rates among sites was set as α = 1 The Dirichlet-gamma prior was set to α = 2 and β = 20 for a diffuse prior. The MCMC was run for 40,000 iterations, with samples taken every other iteration, after a 5% burnin. Two runs were conducted to check for consistency.

We calibrated the phylogeny with the only known fossil from the family, obtained from a 12–24 Myr-old Miocene deposit from northern California. The fossil is an impression that resembles the genus *Lobaria*. A recent study by Cornejo and Scheidegger^37^, used a molecular clock-calibrated phylogeny of *Lobaria*, to estimate the age of the fossil impression and found that it fits into the time frame of *Lobaria* diversification and placed it near the crown of the genus. Based on that, we calibrated the 376 × 96 aa concatenated phylogeny with the impression fossil, by placing it on the branch between *L*. *linita* and the remaining *Lobaria* species, with a range of 12 to 24 million years.

### Accession codes

Genbank BioSample submission: SUB4924261.

## Supplementary information

Supplementary Information
