# Supplementary material for: Multiple historical processes obscure phylogenetic relationships in a taxonomically difficult group (Lobariaceae, Ascomycota)
Source: Sci Rep. 2019 Jun 20;9:8968. doi: 10.1038/s41598-019-45455-x (PMC6586878; doi:10.1038/s41598-019-45455-x)
Supplement: Supplementary file 1 — Supplementary Information [file 41598_2019_45455_MOESM1_ESM.pdf]

Multiple historical processes obscure phylogenetic relationships in a taxonomically difficult group (Lobariaceae, Ascomycota)

Supplementary Information

Todd J. Widhelm[1,2*,] Felix Grewe[3], Jen-Pan Huang[1,4], Joel A. Mercado-Díaz[1], Bernard Goffinet[5], Robert Lücking[6], Bibiana Moncada[7], Roberta Mason-Gamer[2], and H. Thorsten Lumbsch[1]

[1]Field Museum, Science and Education, Chicago, 60605, USA.

[2]University of Illinois at Chicago, Biological Sciences, Chicago, 60607, USA.

[3]Field Museum, Grainger Bioinformatics Center, Chicago, 60605, USA.

[4]Biodiversity Research Center, Academia Sinica, Taipei, Taiwan.

[5]University of Connecticut, Ecology and Evolutionary Biology, Storrs, 06268, USA.

[6]Botanischer Garten und Botanisches Museum, Herbarium, Berlin, 14195, Germany.

[7]Universidad Distrital Francisco José de Caldas, Torre de Laboratorios, Herbario, Bogotá, Colombia, 11021.

*twidhelm@fieldmuseum.org

Table S1. Hybpiper BLASTX assembly statistics.

Name	Reads Mapped	Genes Mapped	Genes with Contigs	Genes with Seqs	Genes at 25%	Genes at 50%	Genes at 75%	Genes at 150%	Paralog Warnings
9930-Lobaria-pulmonaria	317874	400	398	398	398	398	398	0	19
4005-Ricasolia-amplissima	43711	394	378	378	378	375	366	0	10
15904-Sticta-subcaperata	156960	393	385	381	381	380	377	0	18
15903-Sticta-martinii	154510	394	381	380	379	375	370	0	10
15902-Sticta-lacera-livida	115924	393	384	383	383	381	374	0	19
15901-Sticta-latifrons	181353	393	383	382	382	378	374	0	19
15900-Sticta-filix	145328	396	385	384	383	381	376	0	18
15899-Pseudocyphellaria-rufovirescens	105510	393	376	374	373	371	367	0	14
15898-Pseudocyphellaria-montagnei	172716	393	383	383	381	379	375	0	16
15897-Pseudocyphellaria-episticta	76645	394	376	376	376	376	370	0	12
15896-Pseudocyphellaria-disimilis	63765	389	373	373	373	368	357	0	10
15895-Pseudocyphellaria-degelii	106741	392	380	380	380	378	376	0	16
15894-Pseudocyphellaria-crocatagroup	73117	393	375	375	375	373	370	0	15
15893-Pseudocyphellaria-corbettii	68465	390	375	374	373	371	363	0	10
15892-Pseudocyphellaria-chloroluca	140011	390	375	375	374	371	365	0	13
15891-Pseudocyphellaria-carpoloma	73265	390	376	376	376	375	370	0	10
15890-Pseudocyphellaria-billardierei	99794	394	377	376	376	374	369	0	11
15889-Nephroma-australe	58436	387	319	314	313	296	259	0	7
15888-Crocodia-aurata-poculifera	67681	386	370	370	369	367	360	0	13
15887-Pseudocyphellaria-vaccina	87740	390	378	377	377	375	369	0	24
15886-Pseudocyphellaria-sp	121706	391	379	378	377	376	371	0	14
15885-Pseudocyphellaria-hirsuta	49919	387	365	365	365	362	353	0	20
15884-Pseudocyphellaria-sp	69857	390	374	374	374	374	367	0	14
15882-Sticta-cinereoglauca	7929	386	79	78	74	54	32	0	0
15881-Sticta-fuliginosa	22895	386	311	310	307	294	256	0	8
15880-Sticta-fuliginosa	16247	395	244	242	240	214	182	0	2
15879-Sticta-latifrons	40643	387	359	358	358	352	335	0	9
15878-Pseudocyphellaria-pubescens	77434	389	378	377	377	375	368	0	12
15877-Pseudocyphellaria-lividofusa	81644	388	372	372	372	369	363	0	9
15876-Pseudocyphellaria-cinnamomea	40418	390	364	364	362	357	347	0	9
15875-Pseudocyphellaria-affintricata	28950	386	324	324	324	320	310	0	8
15874-Pseudocyphellaria-haywardiosum	27024	381	312	312	312	310	295	0	8

Name	Reads Mapped	Genes Mapped	Genes with Contigs	Genes with Seqs	Genes at 25%	Genes at 50%	Genes at 75%	Genes at 150%	Paralog Warnings
15873-Sticta-scabrosa	140166	390	380	379	379	376	373	0	23
15872-Sticta-sp	70932	392	377	376	376	375	368	0	18
15871-Sticta-caulescens	66202	396	377	376	375	375	363	0	14
15870-Sticta-hypochra	56609	389	372	370	370	365	357	0	13
15869-Pseudocyphellaria-intricata	51376	390	370	369	369	367	359	0	11
15868-Pseudocyphellaria-coriifolia	19578	392	261	258	258	254	232	0	5
15851-Yarrumia-colensoi	74045	392	377	377	377	376	366	0	30
15850-Yarrumia-coronata	92707	393	383	383	382	381	375	0	31
15849-Pseudocyphellaria-flavicans	81891	390	377	376	376	375	365	0	13
15848-Pseudocyphellaria-freycinetii	65127	391	374	374	374	372	366	0	12
15847-Pseudocyphellaria-berberina	95431	391	381	380	380	380	377	0	13
15845-Pseudocyphellaria-crocata	63722	387	372	372	372	372	363	0	12
15844-Pseudocyphellaria-crocata	91425	387	375	375	0	0	0	0	14
15843-Pseudocyphellaria-obvoluta	69568	388	375	375	374	372	366	0	15
15842-Pseudocyphellaria-lecheri	45146	387	367	367	366	365	358	0	12
15841-Pseudocyphellaria-granulata	80330	392	374	373	373	373	369	0	15
15688-Nephroma-sp	37009	381	292	286	284	250	206	0	10
15687-Nephroma-sp	50277	388	306	300	298	275	224	0	8
15686-Lobaria-sp	25823	390	301	299	298	292	266	0	15
15685-Lobaria-sp	26296	390	320	318	318	309	284	0	11
15684-Lobaria-sp	20432	387	253	250	249	243	231	0	7
15683-Ricasolia-sp	59859	390	379	379	379	377	370	0	14
15682-Ricasolia-sp	67366	393	382	382	382	381	371	0	14
15681-Yoshimuriella-sp	43085	390	361	361	361	360	350	0	16
15680-Yoshimuriella-sp	54282	390	374	374	374	371	363	0	19
15679-Yoshimuriella-sp	39663	390	353	352	352	351	337	0	18
15678-Yoshimuriella-sp	23494	390	277	276	274	269	255	0	8
15317-Yarrumia-coronata	78530	395	376	376	376	373	365	0	34
15316-Yarrumia-colensoi	100375	390	381	381	381	379	370	0	28
15313-Crocodia-aurata-poculifera	87348	389	369	369	369	369	362	0	14
15280-Yoshimuriella-peltigera	85126	393	382	381	380	378	371	1	17
15279-Yoshimuriella-peltigera	175762	395	385	385	385	382	374	0	18
15278-Yoshimuriella-subdissecta	167360	393	386	386	386	386	380	1	20

Name	Reads Mapped	Genes Mapped	Genes with Contigs	Genes with Seqs	Genes at 25%	Genes at 50%	Genes at 75%	Genes at 150%	Paralog Warnings
15277-Lobariella-sp	88480	391	385	385	384	380	359	1	17
15276-Lobariella-sp	53711	392	378	378	375	364	339	1	23
15275-Lobariella-sp	89805	393	383	382	382	380	361	0	22
15274-Lobarina-oregana	103892	398	389	389	389	387	383	0	16
15273-Nephroma-plumbeum	43210	381	294	290	287	250	181	0	4
15272-Dendriscosticta-affwrightii	67788	392	383	383	383	383	375	0	13
15271-Anomalobaria-anomala	47259	396	385	384	383	382	379	0	13
15261-Dendriscosticta-sp	76478	392	384	384	384	382	374	0	18
15258-Nephroma-antarcticum	62403	384	341	333	330	297	249	0	6
15257-Lobaria-sp	72323	397	395	395	395	393	392	0	16
15256-Lobaria-sp	91578	399	398	398	398	398	396	0	18
15254-Lobaria-linita	69494	397	392	392	392	392	390	0	16
15253-Dendriscosticta-kurokawae	84925	392	386	385	385	383	374	0	18
15156-Sticta-sp	34842	384	344	343	339	332	307	0	8
15155-Sticta-sp	31980	392	344	343	339	329	301	0	9
15144-Pseudocyphellaria-homoeophylla	84774	394	377	376	376	375	368	0	14
14850-Pseudocyphellaria-rubrina	212630	396	387	387	386	385	381	0	29
14845-Pseudocyphellaria-granulata	70414	390	376	376	376	375	371	0	14
14840-Pseudocyphellaria-neglecta	87835	389	374	374	374	373	371	0	15
14835-Pseudocyphellaria-crocata	52660	387	367	367	367	365	359	0	11
14830-Pseudocyphellaria-billardierei	82895	391	378	376	376	374	372	0	12
14829-Pseudocyphellaria-dissimilis	34235	388	348	348	348	347	333	0	10
14681-Pseudocyphellaria-glabra	169690	395	380	379	379	378	372	0	15
14665-Sticta-densiphyllidata	63900	392	378	377	377	374	362	0	16
14657-Sticta-affsublimbatoides	12791	381	199	195	194	179	143	0	2
14538-Sticta-scabrosa	104206	393	380	379	379	376	370	0	15
14532-Sticta-weigelii	30708	387	345	345	345	338	315	0	11
14493-Sticta-carolinensis	119992	390	383	380	380	380	376	0	17
14492-Sticta-beauvoisii	165286	394	381	380	380	379	376	0	15
10049-Sticta-afflimbata	37025	390	352	351	350	342	334	0	11
10048-Sticta-afffuliginosa	36811	384	349	349	347	343	333	0	13

Table S2. Samples sequenced using target capture in the current study with voucher data and Genbank accession numbers.

DNA #	*Genus*	*Species*	Collector	Voucher	Herbarium	Country	Genbank #
4005	*Ricasolia*	*amplissima*	Tønsberg	44719	F	Norway	SAMN10602979
9930	*Lobaria*	*pulmonaria*	Widhelm	TW4	F	U.S.A	SAMN10602980
10048	*Sticta*	*aff. fuliginosa*	McCune	35727	F	U.S.A	SAMN10602981
10049	*Sticta*	*aff. limbata*	McCune	35726	F	U.S.A	SAMN10602982
14492	*Sticta*	*beauvoisii*	Taylor Quedensely	*TQ16699*	F	U.S.A	SAMN10602983
14493	*Sticta*	*carolinensis*	Taylor Quedensely	*TQ16700*	F	U.S.A	SAMN10602984
14532	*Sticta*	*weigelii*	Joel Mercado-Diaz	2284	F	Puerto Rico	SAMN10602985
14538	*Sticta*	*scabrosa*	Joel Mercado-Diaz	2287	F	Puerto Rico	SAMN10602986
14657	*Sticta*	*aff. sublimbatoides*	Joel Mercado-Diaz	2378	F	Puerto Rico	SAMN10602987
14665	*Sticta*	*densiphyllidata*	Joel Mercado-Diaz	2389	F	Puerto Rico	SAMN10602988
14681	*Pseudocyphellaria*	*glabra*	Lumbsch, Widhelm & Grewe	2046 A	F	Australia	SAMN10602989
14829	*Pseudocyphellaria*	*dissimilis*	Lumbsch, Widhelm & Grewe	2173	F	Australia	SAMN10602990
14830	*Pseudocyphellaria*	*carpoloma*	Lumbsch, Widhelm & Grewe	2074 D	F	Australia	SAMN10602991
14835	*Pseudocyphellaria*	*crocata*	Lumbsch, Widhelm & Grewe	2145	F	Australia	SAMN10602992
14840	*Pseudocyphellaria*	*neglecta*	Lumbsch, Widhelm & Grewe	2031	F	Australia	SAMN10602993
14845	*Pseudocyphellaria*	*granulata*	Lumbsch, Widhelm & Grewe	2469	F	Australia	SAMN10602994
14850	*Yarrumia*	*coronata*	Lumbsch, Widhelm & Grewe	2042	F	Australia	SAMN10602995
15144	*Pseudocyphellaria*	*homoeophylla*	de Lange, Lucking & Moncada	38131	F	New Zealand	SAMN10602996
15155	*Sticta*	*sp.*	Coca	43	F	Colombia	SAMN10602997
15156	*Sticta*	*sp.*	Coca	47	F	Colombia	SAMN10602998
15253	*Dendriscosticta*	*platyphylla*	Goffinet	13475	CONN	Taiwan	SAMN10602999
15254	*Lobaria*	*linita*	Simon	147	CONN	Canada	SAMN10603000
15256	*Lobaria*	*sp.*	Goffinet	13511	CONN	Taiwan	SAMN10603001
15257	*Lobaria*	*sp*	Goffinet	13563	CONN	Taiwan	SAMN10603002
15258	*Nephroma*	*antarcticum*	Goffinet	13963	CONN	Chile	SAMN10603003
15261	*Ricasolia*	*sp.*	Goffinet	13062	CONN	Taiwan	SAMN10603004
15271	*Anomalobaria*	*anomala*	Simon	150	CONN	Canada	SAMN10603005
15272	*Dendriscosticta*	*aff. wrightii*	Goffinet	13227	CONN	Taiwan	SAMN10603006
15273	*Nephroma*	*plumbeum*	Goffinet	12673	CONN	Chile	SAMN10603007

DNA #	*Genus*	*Species*	Collector	Voucher	Herbarium	Country	Genbank #
15274	*Lobarina*	*oregana*	Simon	143	CONN	Canada	SAMN10603008
15275	*Lobariella*	*sp.*	Lucking	41013a	BGBM	Colombia	SAMN10603009
15276	*Lobariella*	*sp.*	Lucking	41042	BGBM	Colombia	SAMN10603010
15277	*Lobariella*	*sp.*	Lucking	41025	BGBM	Colombia	SAMN10603011
15278	*Yoshimuriella*	*subdissecta*	Moncada	5369	BGBM	Colombia	SAMN10603012
15279	*Yoshimuriella*	*peltigera*	Moncada	5389	BGBM	Colombia	SAMN10603013
15280	*Yoshimuriella*	*peltigera*	Moncada	5322	BGBM	Colombia	SAMN10603014
15313	*Crocodia*	*poculifera*	de Lange, Lucking & Moncada	39102	F	New Zealand	SAMN10603015
15316	*Yarrumia*	*colensoi*	de Lange, Lucking & Moncada	38741	F	New Zealand	SAMN10603016
15317	*Yarrumia*	*coronata*	de Lange, Lucking & Moncada	38974	F	New Zealand	SAMN10603017
15678	*Yoshimuriella*	*sp.*	Joel Mercado-Diaz	2955	F	Dominican Republic	SAMN10603018
15679	*Yoshimuriella*	*sp.*	Joel Mercado-Diaz	3072i	F	Dominican Republic	SAMN10603019
15680	*Yoshimuriella*	*sp.*	Joel Mercado-Diaz	3067b	F	Dominican Republic	SAMN10603020
15681	*Yoshimuriella*	*sp.*	Joel Mercado-Diaz	3133b	F	Dominican Republic	SAMN10603021
15682	*Ricasolia*	*sp.*	Joel Mercado-Diaz	2939	F	Dominican Republic	SAMN10603022
15683	*Ricasolia*	*sp.*	Joel Mercado-Diaz	3031	F	Dominican Republic	SAMN10603023
15684	*Yoshimuriella*	*sp.*	Joel Mercado-Diaz	2991a	F	Dominican Republic	SAMN10603024
15685	*Ricasolia*	*sp.*	Joel Mercado-Diaz	3038c	F	Dominican Republic	SAMN10603025
15686	*Lobariella*	*sp.*	Joel Mercado-Diaz	3063a	F	Dominican Republic	SAMN10603026
15687	*Nephroma*	*sp.*	Joel Mercado-Diaz	3122f	F	Dominican Republic	SAMN10603027
15688	*Nephroma*	*sp.*	Joel Mercado-Diaz	3104	F	Dominican Republic	SAMN10603028
15841	*Pseudocyphellaria*	*granulata*	Felix Grewe, Todd Widhelm, Matt von Konrat, Juan Larrain	4117	F	Chile	SAMN10603029
15842	*Pseudocyphellaria*	*lechleri*	Felix Grewe, Todd Widhelm, Matt von Konrat, Juan Larrain	4118	F	Chile	SAMN10603030
15843	*Parmostictina*	*obvoluta*	Felix Grewe, Todd Widhelm, Matt von Konrat, Juan Larrain	4119	F	Chile	SAMN10603031
15844	*Pseudocyphellaria*	*crocata*	Felix Grewe, Todd Widhelm, Matt von Konrat, Juan Larrain	4121	F	Chile	SAMN10603032
15845	*Pseudocyphellaria*	*crocata*	Felix Grewe, Todd Widhelm, Matt von Konrat, Juan Larrain	4122	F	Chile	SAMN10603033
15847	*Podostictina*	*berberina*	Felix Grewe, Todd Widhelm, Matt von Konrat, Juan Larrain	4124	F	Chile	SAMN10603034
15848	*Pseudocyphellaria*	*freycinetii*	Felix Grewe, Todd Widhelm, Matt von Konrat, Juan Larrain	4125	F	Chile	SAMN10603035
15849	*Podostictina*	*flavicans*	Todd Widhelm, Matt von Konrat, Juan Larrain	4389	F	Chile	SAMN10603036
15850	*Yarrumia*	*coronata*	de Lange, Lucking & Moncada	38675	F	New Zealand	SAMN10603037

DNA #	*Genus*	*Species*	Collector	Voucher	Herbarium	Country	Genbank #
15851	*Yarrumia*	*colensoi*	de Lange, Lucking & Moncada	38836	F	New Zealand	SAMN10603038
15868	*Pseudocyphellaria*	*coriifolia*	Todd Widhelm, Matt von Konrat, Juan Larrain	4319	F	Chile	SAMN10603039
15869	*Pseudocyphellaria*	*intricata*	Todd Widhelm, Matt von Konrat, Juan Larrain	4353	F	Chile	SAMN10603040
15870	*Sticta*	*hypochroa*	Todd Widhelm, Matt von Konrat, Juan Larrain	4354	F	Chile	SAMN10603041
15871	*Sticta*	*caulescens*	Todd Widhelm, Matt von Konrat, Juan Larrain	4357	F	Chile	SAMN10603042
15872	*Sticta*	*sp.*	Todd Widhelm, Matt von Konrat, Juan Larrain	4358	F	Chile	SAMN10603043
15873	*Podostictina*	*encoensis*	Todd Widhelm, Matt von Konrat, Juan Larrain	4401	F	Chile	SAMN10603044
15874	*Pseudocyphellaria*	*haywardiosum*	Peter de Lange	12586	F	New Zealand	SAMN10603045
15875	*Pseudocyphellaria*	*haywardiosum*	Peter de Lange	12587	F	New Zealand	SAMN10603046
15876	*Pseudocyphellaria*	*cinnamomea*	Mark Moorhouse	UNITEC 10400	F	New Zealand	SAMN10603047
15877	*Pseudocyphellaria*	*lividofusa*	Peter de Lange	13259	F	New Zealand	SAMN10603048
15878	*Pseudocyphellaria*	*pubescens*	Mark Moorhouse	UNITEC 10413	F	New Zealand	SAMN10603049
15879	*Sticta*	*latifrons*	Peter de Lange	CH2534	F	New Zealand	SAMN10603050
15880	*Sticta*	*fuliginosa*	Peter de Lange	CH2547	F	New Zealand	SAMN10603051
15881	*Sticta*	*fuliginosa*	Peter de Lange	13163	F	New Zealand	SAMN10603052
15882	*Sticta*	*cinereoglauca*	Peter de Lange	CH2547	F	New Zealand	SAMN10603053
15884	*Pseudocyphellaria*	*sp.*	Todd Widhelm, Matt von Konrat, Juan Larrain	4405	F	Chile	SAMN10603054
15885	*Parmostictina*	*hirsuta*	Todd Widhelm, Matt von Konrat, Juan Larrain	4417	F	Chile	SAMN10603055
15886	*Pseudocyphellaria*	*sp.*	Todd Widhelm, Matt von Konrat, Juan Larrain	4440	F	Chile	SAMN10603056
15887	*Podostictina*	*vaccina*	Todd Widhelm, Matt von Konrat, Juan Larrain	4443	F	Chile	SAMN10603057
15888	*Crocodia*	*aurata*	Felix Grewe, Todd Widhelm	3496	F	New Zealand	SAMN10603058
15889	*Nephroma*	*australe*	Felix Grewe, Todd Widhelm	3516	F	New Zealand	SAMN10603059
15890	*Pseudocyphellaria*	*carpoloma*	Felix Grewe, Todd Widhelm	3192	F	New Zealand	SAMN10603060
15891	*Pseudocyphellaria*	*carpoloma*	Felix Grewe, Todd Widhelm	3617	F	New Zealand	SAMN10603061
15892	*Pseudocyphellaria*	*chloroluca*	Felix Grewe, Todd Widhelm, Dan Blanchon, Peter de Lange	3044	F	New Zealand	SAMN10603062
15893	*Pseudocyphellaria*	*corbettii*	Felix Grewe, Todd Widhelm	3190	F	New Zealand	SAMN10603063
15894	*Pseudocyphellaria*	*crocata*	Allison Knight	69193a	OTA	New Zealand	SAMN10603064
15895	*Podostictina*	*degelii*	Felix Grewe, Todd Widhelm	3594	F	New Zealand	SAMN10603065
15896	*Pseudocyphellaria*	*disimilis*	Felix Grewe, Todd Widhelm	3019	F	New Zealand	SAMN10603066
15897	*Pseudocyphellaria*	*episticta*	Felix Grewe, Todd Widhelm	3498	F	New Zealand	SAMN10603067

DNA #	***Genus***	***Species***	Collector	Voucher	Herbarium	Country	Genbank #
15898	*Pseudocyphellaria*	*montagnei*	Felix Grewe, Todd Widhelm	3507	F	New Zealand	SAMN10603068
15899	*Pseudocyphellaria*	*rufovirescens*	Felix Grewe, Todd Widhelm	3219	F	New Zealand	SAMN10603069
15900	*Sticta*	*filix*	Felix Grewe, Todd Widhelm, Dan Blanchon, Peter de Lange	3074	F	New Zealand	SAMN10603070
15901	*Sticta*	*latifrons*	Felix Grewe, Todd Widhelm	3274	F	New Zealand	SAMN10603071
15902	*Sticta*	*lacera*	Felix Grewe, Todd Widhelm	3255	F	New Zealand	SAMN10603072
15903	*Pseudocyphellaria*	*corbettii*	Felix Grewe, Todd Widhelm, Dan Blanchon, Peter de Lange	3105	F	New Zealand	SAMN10603073
15904	*Sticta*	*subcaperata*	Felix Grewe, Todd Widhelm	3310	F	New Zealand	SAMN10603074

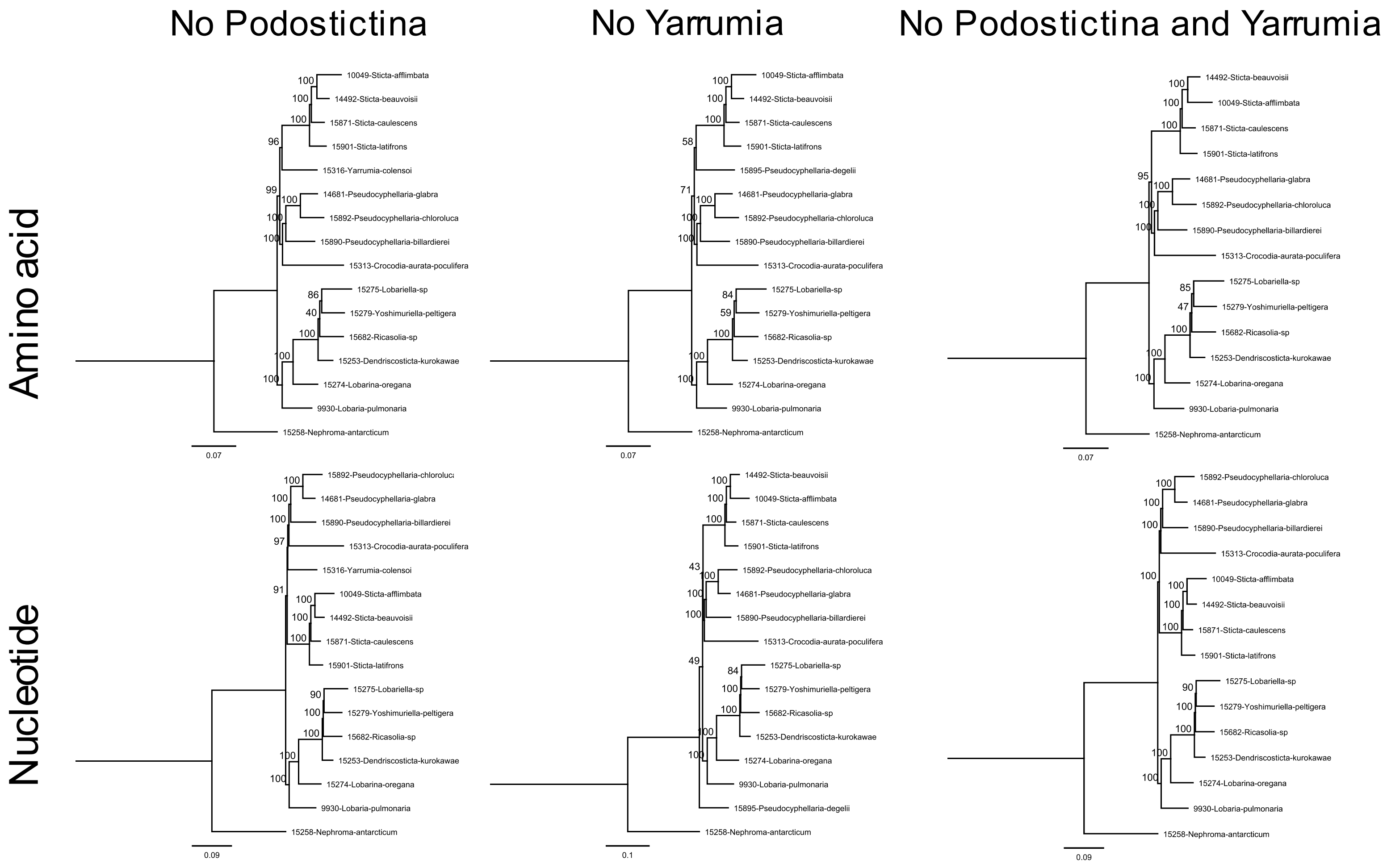

Figure S1. Maximum likelihood analyses conducted without *Podostictina*, *Yarrumia*, or both. Bootstrap support is depicted at the nodes.

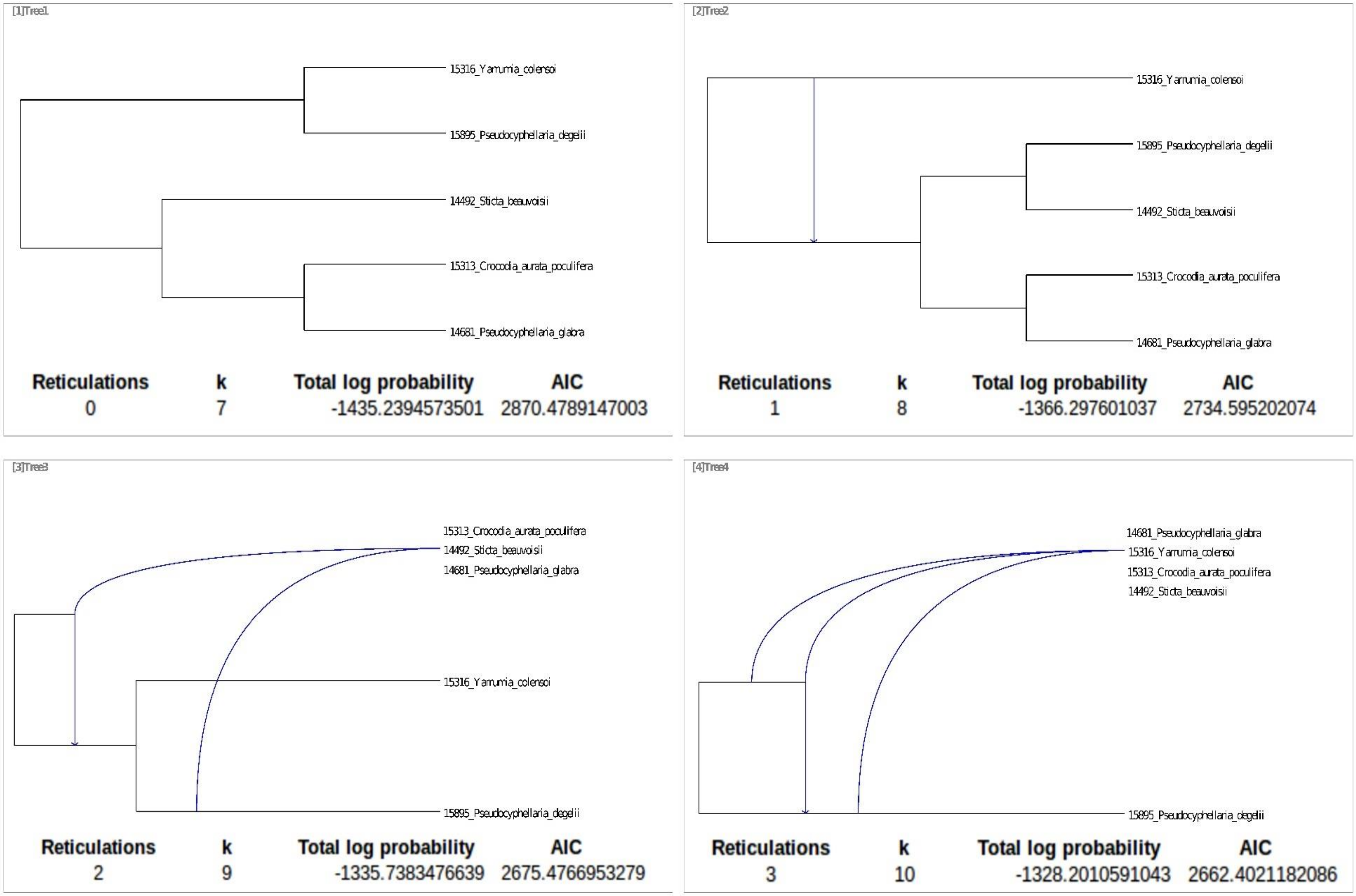

Figure S2. PhyloNet ML networks for five taxa. For each reticulation scenario, the log likelihood and AIC are reported under the networks.

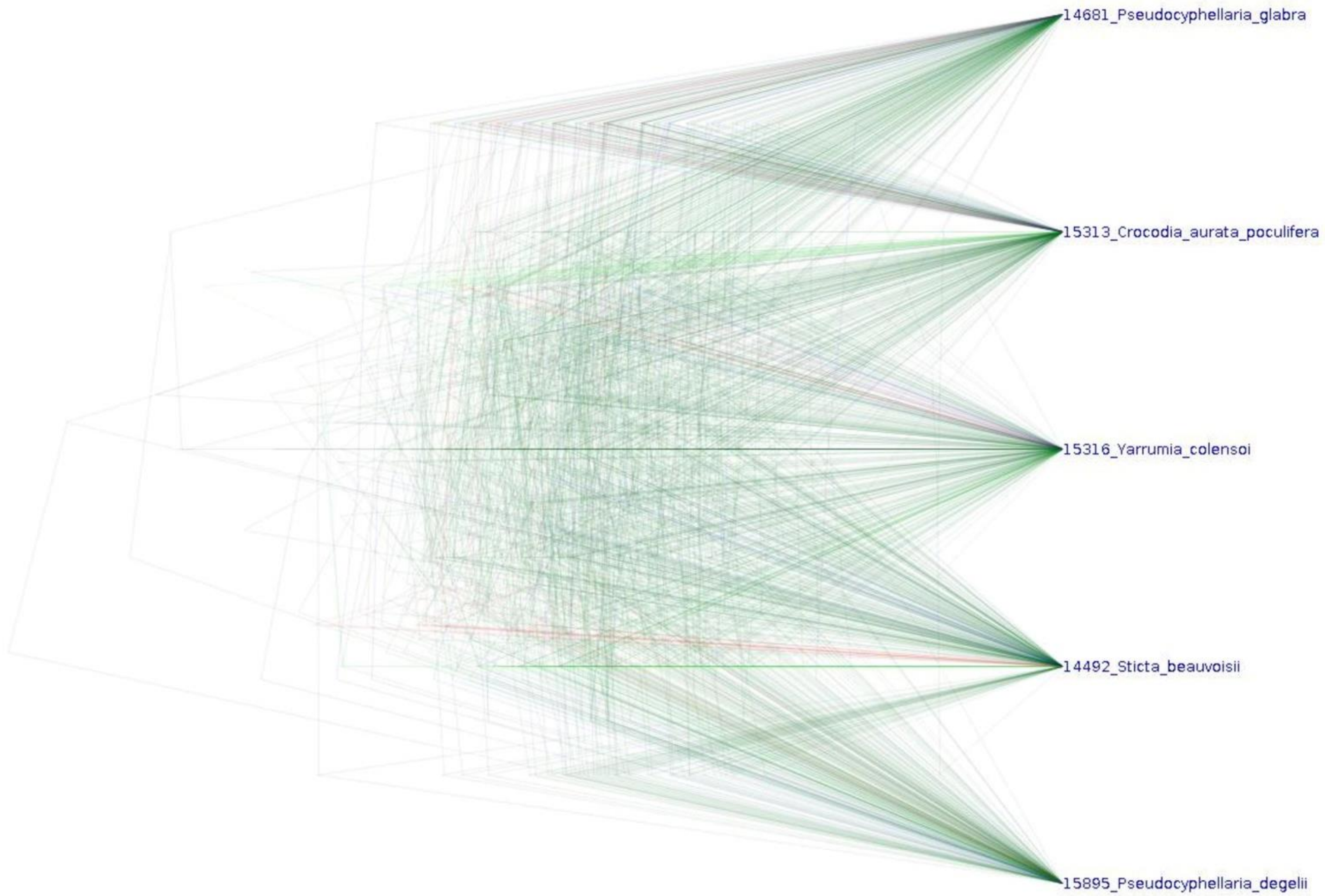

Figure S3. A DensiTree plot of 297 gene trees used to produce the five-taxa datasets
